# The Week's Work

**Published:** 1910-12-10

**Authors:** 


### The Week's Work.

### THE IDEAL WARD FLOOR.

In the interesting "Report on Hospital Construction," the committee appointed by the American Hospitals Association has gone fully into questions of outstanding interest to hospital architects, and the *résumé* of the paper, which appears in the current issue of the *International Hospital Record*, shows that the committee has considered various aspects of several important points in connection with the planning of hospital wards. Superintendent Webster, of the Royal Victoria Hospital, Montreal, who is responsible for the report, discusses the entire problem of how a hospital should be designed and built. His remarks are very instructive reading, and his paper contains many valuable tips which are worth noting. For instance, the old, old question, "What flooring proves the best for a ward?" is answered after an experience of nearly every imaginable variety of floor. At the Royal Victoria they have "nine different kinds of flooring—and still no perfect floor." In that institution experiments have been made with the new "asbestos board flooring," which is well spoken of in Continental private clinics, with ordinary plain concrete, with hardwood, terrazzo mosaic, Tennessee tile, cork, slate, hardwood, doloment cement composition, and all varieties of clay tiles. In the circumstances the committee's finding is very useful to those who have not had the opportunity of such extensive practical experience with the several kinds of flooring. Mr. Webster strongly urges the claims of the hardwood floor, made of well-seasoned maple. With the opinion that this is "the finest floor yet produced for wards, and one that can with care be laid perfectly" most people who have had any experience of this type of flooring will readily agree. A few practical points are raised in the report in reference to the construction and upkeep of these maple floors.

### THE MAPLE FLOOR.

"A good deal of the imperfections," writes Mr. Webster, "and open joints are from the wood not being dry and from it having been improperly laid. Those proposing to build should, at the outset, purchase the hardwood to be used and store it, thus ensuring dry and properly seasoned wood for the floors when the time comes for them to be laid. The wood should always be laid running in the travel of the ward." He suggests the following treatment for such a floor: "New floors treat with oil, then shellac, and afterwards two coats of varnish (Marnot). When thoroughly dry, wax with the following preparation: Beeswax $\frac{1}{2}$ lb., 1 oz. rosin, 1 oz. pulv. alum, 2 pints turpentine, 1 tablespoonful Japan (dryer) turpentine. Mix the wax and rosin over a fire, then remove from the heat, add your turpentine and Japan, and your alum last. Then let it stand till it forms a paste. When the floor is first varnished it takes three pounds of this mixture for one of our large ward floors. . . . I strongly advise all superintendents to make their own preparation at the hospital, thus saving money and good floors. Apply the wax with waste and then brush. We use seventy pounds of this wax preparation in all a year; I simply mention this fact because I have been astounded at the large quantities bought by some hospitals. . . . All hospitals should possess a Simplex electric planer and sandpaper polisher, which will remove the old varnish and bring the floor 'back new.' Another method, if the machine is not available, is to apply a preparation of Gillett's lye and hot water, and then sandpaper: this removes the varnish and smooths the floor." For the operating theatre Mr. Webster recommends the Tennessee tile, which provides a smooth surface owing to the flush jointing. He also favours cork composition laid with a liquid cement; doloment composition is fireproof and fairly satisfactory, but very liable to crack.

### FURTHER POINTS FROM THE REPORT.

Six years ago the Royal Victoria Hospital was damaged by a fire, which broke out in the administrative block, and in rebuilding the "trustees resolved to fireproof the whole institution from floor to ceiling. In doing this," remarks Mr. Webster, "we kept in mind the object of lessening in every way the possibility of noise penetrating from one floor or room to another. The material chosen for the work was the usual steel, concrete, and terra-cotta with expanded metal, and nothing tends to cause less echo and noise than this class of material. In laying all the floors we used steel and concrete six inches thick, with sleepers to receive the hard maple or finished floor, and on the sleepers we laid asbestos sheeting over the whole, then the finished maple floor, making these floors almost absolutely soundproof as well as fireproof. All the rough walls, before receiving the terra-cotta or metal lining, were sprayed with a mixture of lime before either plastering or flooring, making the work clean and vermin-proof. By using tin or aluminium sheeting between the floors or walls you can make the rooms absolutely soundproof." It is worth while, also, to notice that the report lays stress on the advisability of using the best materials, and to build for the future. These are points on which we have always insisted. They are, unfortunately, too often overlooked by designers and hospital architects, and it is well that they should be constantly held in view if the best possible results are to be obtained in the construction of new institutions. We hope to deal with the report in a future issue at greater length.

### HOSPITALS AND THEIR MEDICAL SCHOOLS.

We have already given in recent numbers (The Hospital, pp. 265-266, 299) the letters written by the Metropolitan Radical Federation, and the views of the King's Fund and St. George's Hospital in answer to these letters. The question raised is one of the deepest importance, not only to the hospitals, as charities to relieve the distress of the poor and needy, but to the welfare and the future of the medical schools of such institutions as have medical students attached to them. When the Fry Commission in 1905 laid down the dictum that the obligations of the school to the hospital and the hospital to the school balanced themselves, it seemed simple to carry out such a just ruling, and few would have realised how difficult it would be to define the line of demarcation between the two conflicting interests. How far the school can do the work of the hospital, and the hospital supply the needs of the school, must ever be in dispute; and this all the more renders necessary some authority to decide the knotty point. If the school of St. George's Hospital does the pathological work of the hospital it must be paid for the service, but whether the hospital is justified in paying the fee debited to the charity account must, until there is some central controlling body, be left to the good judgment of the hospital Board. Without attempting, however, to judge between two opinions so sharply opposed, one cannot help being struck by the similarity of argument put forward by the Metropolitan Radical Federation and the National Anti-Vivisection Society. In Mr. Garrity's letter are reproduced most of the facts and figures printed in red in Mr. Coleridge's "Guide to the Charitable." Every year, about the middle of December, this publication is sent broadcast, just at the moment when the public is most generously disposed towards the hospitals. This coincidence, if coincidence it be, raises a side issue that is likely to be prejudicial to the hospitals. The public, however, may be assured that it can leave the just administration of the financial relations between hospitals and schools in the hands of those who for so many years have administered the great funds.

### AUSTRALIAN HOSPITALS AND OLD AGE PENSIONERS.

From an editorial in the *Prince Alfred Hospital Gazette*, of Sydney, it would appear that in Australia old age pensioners, when they become hospital patients, are a charge wholly on the hospital funds, whilst the Commonwealth is, partially at least, relieved of the payment of pensions. The Executive of this hospital has taken action in urging the necessity for a revision of this policy of the Federal Parliament, and has communicated with the authorities of all the general hospitals in the State urging that an appeal should be addressed to the Federal Government to alter the system so that at least 5s. per week should be paid to hospitals on behalf of old age pensioners while they are in hospital, and that the funerals of such patients, when they died in hospital, should be paid for by the Commonwealth, instead of by the hospitals, as at present. The response to this appeal has been practically unanimous, almost every hospital writing to the Minister, or appealing to him through the local member, to make the alteration suggested. It is said that the Federal Government has the matter under favourable consideration at present.

### THE ROYAL HOSPITAL FOR INCURABLES.

We are never tired of drawing attention in our columns to the value of making hospital reports readable, and what is a necessity for the success of a report is the first necessity for the success of an appeal. This week we are happy to be able to point to a capital example, namely, the appeal just issued by the Royal Hospital for Incurables, Putney Heath. The appeal consists of an eight-page pamphlet containing an extract from a speech on behalf of this hospital which Charles Dickens made so long ago as 1857. That the speech is still fresh and makes capital reading proves what a good journalist Charles Dickens was; and the Royal Hospital for Incurables has shown itself quick to recognise its value. This speech, no doubt, was unearthed from the archives of the institution, and though it would perhaps be going too far to say that a search in the archives of every hospital would reveal an unpublished speech by Charles Dickens, a rummage through old papers would prove more frequently than not an inspiration to hospital secretaries in want of an idea for attracting public support. This is the moral which we venture to draw from the Royal Hospital for Incurables' pamphlet, and the institution is to be congratulated on producing an appeal of real charm, novelty, and value.

### THIS WEEK'S DRUG MARKET.

A steady business in drugs continues to be done, and most of the changes in value which have taken place are in an upward direction. Ergot of rye continues to advance in price, and it seems not improbable that much higher figures will be reached The price of gum benzoin is well maintained, and makers of benzoic acid, ammonium benzoate, and other benzoates have raised their quotations. Eserine has been advanced in price owing to the scarcity of Calabar beans. Oil of sandalwood is dearer. Higher prices are expected for olive oil owing to the poorness of the olive crops in practically all countries. Opium still exhibits a dearer tendency. Castor oil is advancing in value owing to the scarcity of seed. Essence of lemon is quoted a few pence per lb. higher. Calumba root is dearer, and Saffron continues to advance in price. Coca leaves are also dearer. A further increase in the cost of cocaine is not altogether improbable. Manna is tending higher in price. Tartaric acid is again quoted at a slightly higher figure. Rhubarb is still selling at low rates, but it is not unlikely that prices will recover.

